# A SARS-CoV-2 Wuhan spike virosome vaccine induces superior neutralization breadth compared to one using the Beta spike

**DOI:** 10.1038/s41598-022-07590-w

**Published:** 2022-03-10

**Authors:** Yme U. van der Velden, Marloes Grobben, Tom G. Caniels, Judith A. Burger, Meliawati Poniman, Melissa Oomen, Esther Siteur-van Rijnstra, Khadija Tejjani, Denise Guerra, Ronald Kempers, Toon Stegmann, Marit J. van Gils, Rogier W. Sanders

**Affiliations:** 1 Department of Medical Microbiology and Infection Prevention, Amsterdam UMC, Amsterdam Institute for Infection and Immunity, University of Amsterdam, 1105 AZ Amsterdam, The Netherlands; 2 Experimental Immunology, Amsterdam UMC, Amsterdam Institute for Infection and Immunity, University of Amsterdam, 1105 AZ Amsterdam, The Netherlands; 3 Mymetics BV, JH Oortweg 21, 2333 CH Leiden, The Netherlands; 4 Department of Microbiology and Immunology, Weill Medical College of Cornell University, 1300 York Avenue, New York, NY 10065 USA

**Keywords:** Vaccines, Protein vaccines, Infectious diseases

## Abstract

Current SARS-CoV-2 vaccines are effective, but long-term protection is threatened by the emergence of virus variants. We generated a virosome vaccine containing the Beta spike protein and compared its immunogenicity in mice to a virosome vaccine containing the original Wuhan spike. Two administrations of the virosomes induced potent SARS-CoV-2 neutralizing antibodies in both vaccine groups. The level of autologous neutralization in Beta-vaccinated mice was similar to the level of autologous neutralization in Wuhan-vaccinated mice. However, heterologous neutralization to the Wuhan strain in Beta-vaccinated mice was 4.7-fold lower than autologous neutralization, whereas heterologous neutralization to the Beta strain in Wuhan-vaccinated mice was reduced by only 1.9-fold compared to autologous neutralization levels. In addition, neutralizing activity against the D614G, Alpha and Delta variants was also significantly lower after Beta spike vaccination than after Wuhan spike vaccination. Our results show that Beta spike vaccination induces inferior neutralization breadth. These results are informative for programs aimed to develop broadly active SARS-CoV-2 vaccines.

## Introduction

With over 240 million confirmed infections and more than four million deaths as of October 2021, the COVID-19 pandemic continues to disrupt human activity worldwide^1^. While a number of effective vaccines have been approved and are being rolled out, the continuous emergence of new genetic and antigenic SARS-CoV-2 virus variants poses a threat to the long-term effectiveness of these vaccines. The WHO has defined four variants-of-concern (VOCs) based on suspected increased transmissibility, increased pathogenicity and decreased effectiveness of vaccines^2^. These VOCs include Alpha (B.1.1.7), Beta (B.1.351), Gamma (P.1), Delta (B.1.617.2) and since recently omicron (B.1.1.529)^3–7^. Many studies have shown that sera from convalescent individuals and vaccinees have reduced neutralizing activity against in particular Beta and Delta^8–15^. Furthermore, several vaccines were less efficacious against VOCs than the original virus in field trials^16–24^. In fact, the AstraZeneca (AZD1222) vaccine was virtually ineffective in South Africa when Beta dominated^20^. As a consequence, several vaccine companies announced that they were working on new and/or updated vaccines based on VOCs, specifically Beta and Delta. Researchers from Moderna have reported on the immunogenicity of a Beta version of their mRNA vaccine (mRNA-1273) in mice and humans^25, 26^. These efforts are now overtaken by the recent emergence of Omicron and research on updating vaccines has shifted to the more antigenically distinct Omicron variant^27^.

The vast majority of SARS-CoV-2 vaccines are based on the spike (S) glycoprotein, a homotrimeric glycoprotein that plays a pivotal role in viral entry and consists of an S1 subunit including the receptor binding domain (RBD) and an S2 subunit containing the fusion peptide^28, 29^. The RBD is the most prominent target for neutralizing antibodies although other domains can also be targeted. The S proteins in most licensed vaccines contain a double proline mutation intended to stabilize S in its prefusion form, a strategy that originated from HIV-1 vaccine research^30, 31^.

Virosomes are the reconstituted membranes of influenza virus, forming 100–150 nm particles, made of a lipid bilayer membrane containing the influenza hemagglutinin and neuraminidase glycoproteins^32, 33^. These particles can be modified to multivalently display an antigen of choice. While the influenza components can augment antibody responses by exploiting intrastructural help through T cells^34, 35^, virosomes also allow for the specific incorporation of adjuvants of choice^36^, thereby tailoring the immune responses to a given antigen. Approximately 80 million people have been vaccinated with licensed virosome vaccines, Inflexal® against influenza virus and Epaxal® against hepatitis A virus, that have excellent tolerability and safety profiles in children, adults, as well as immunocompromised and chronically ill individuals^37–40^. Furthermore, virosome vaccines for HIV-1 and malaria have reached clinical phase testing^41–43^. The virosome platform appears well-suited to present SARS-CoV-2 spike proteins and warrants the development of a virosome-based SARS-CoV-2 vaccine.

**Figure 1:**
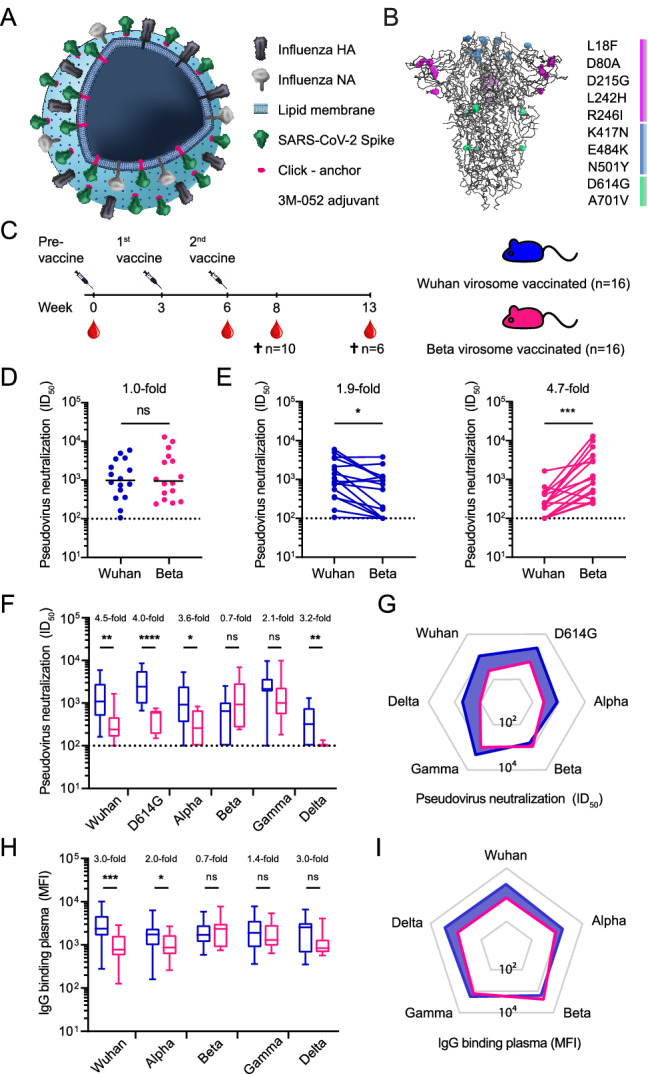
Binding and neutralizing antibody responses elicited by Wuhan and Beta spike virosome vaccines. (**A**) Schematic representation of a virosome with coupled SARS-CoV-2 spike protein. (**B**) SARS-CoV-2 spike structure, with the amino acid mutations in the Beta spike highlighted, compared to the parental Wuhan spike. Amino acid mutations in the N-terminal domain are indicated in pink, mutations in the receptor binding domain in blue and mutations in the S2 domain in green^45^. The beta immunogen contains the L242H and R246I substitutions that were present in early Beta strains, and not the the 242–244 deletion that later became dominant in the beta lineage^45^. (**C**) Vaccination schedule. Blood droplets indicate the weeks at which blood was collected. In all subfigures, Wuhan-vaccinated animals are depicted in blue and Beta-vaccinated animals in magenta. Crosses indicate time of sacrifice for the indicated number of animals. The pre-vaccine is a virosome vaccine without spike protein. (**D**) Autologous SARS-CoV-2 pseudovirus neutralization titers at week 8 (n = 16 per group) for Wuhan pseudovirus and Beta pseudovirus. (**E**) Paired comparison of autologous and heterologous SARS-CoV-2 pseudovirus neutralization titers for Wuhan pseudovirus and Beta pseudovirus at week 8 (n = 16 per group). (**F**) SARS-CoV-2 pseudovirus neutralization titers against Wuhan, D614G and all variants of concern at week 8 (n = 16 per group). Median and 25th to 75th percentiles are indicated by the box, whiskers indicate the minimum and maximum value. (**G**) Spiderweb representation of the median ID_50_ values from (**F**). (**H**) Anti-SARS-CoV-2 spike IgG levels in plasma measured by Luminex assay using beads coated with spike proteins of Wuhan and all variants of concern at week 8 (n = 16 per group). Median and 25th to 75th percentiles are indicated by the box, whiskers indicate the minimum and maximum value. (**I**) Spiderweb representation of the median IgG levels from (**H**). Mann–Whitney U-tests were used for unpaired comparisons and Wilcoxon matched-pairs signed rank test for paired comparisons (* = *p* < 0.05; ** = *p* < 0.01; *** = *p* < 0.001; **** = *p* < 0.0001, ns = not significant). The dotted lines are the assay cut-off for the pseudovirus neutralization assay. HA = hemagglutinin, NA = neuraminidase.

We generated virosome vaccines using the SARS-CoV-2 Beta and original Wuhan spike proteins as immunogens. Virosomes were produced from detergent-solubilized influenza virus membranes to which a lipid head-group modified for click chemistry (DBCO-PE) was added. The spike proteins of both Beta and Wuhan strains (Fig. 1A + B) were conjugated to the virosomes via azide-DBCO click chemistry followed by addition of the TLR7/8 agonist adjuvant 3M-052 to the virosome membrane^44^. To mimic an influenza-experienced immune system, all animals were pre-vaccinated with inactivated influenza virus. Three weeks later one group of animals (n = 16) received a subcutaneous injection of Beta virosomes, while a second group (n = 16) received Wuhan virosomes (Fig. 1C). These injections were repeated after three weeks. Ten animals of each group were sacrificed two weeks after the second vaccination (week 8), while six animals were sacrificed at week 13 to study the durability of the humoral response. None of the mice harbored SARS-CoV-2-specific binding and/or neutralizing antibodies before vaccination (Fig. S1A + B).

Potent neutralizing responses were detected two weeks after the second vaccination in a pseudovirus-based neutralization assay (Fig. 1D). Both groups neutralized autologous pseudovirus equally well (median ID_50_ of 978 and 941 respectively; Fig. 1D). There was no difference between the neutralization levels two or seven weeks after vaccination in either group (Fig. S1C), showing that the virosomes induce neutralizing antibody responses for at least seven weeks.

To assess cross-neutralizing responses, we first determined the capacity of Wuhan virosome-vaccinated mice to neutralize Beta pseudovirus and the capacity of Beta virosome-vaccinated mice to neutralize Wuhan pseudovirus two weeks after the second vaccination. The neutralizing activity of Wuhan virosome-vaccinated mice against Beta was 1.9-fold reduced (median ID_50_ of 648, *p* = 0.02; Fig. 1E). This reduction of plasma neutralization activity against Beta is consistent with results in vaccinated and convalescent human subjects^20, 45, 46^. The neutralizing activity against Wuhan pseudovirus was reduced by 4.7-fold in Beta virosome-vaccinated mice (median ID_50_ of 229, *p* = 0.0003; Fig. 1E). Thus, Wuhan and Beta S virosomes elicited similar levels of neutralization against the matched pseudovirus, while neutralization against the mismatched virus was reduced in both groups, albeit more prominently in Beta virosome-vaccinated mice.

To assess whether the lower heterologous neutralization activity in Beta virosome-vaccinated mice indicates a generalized lower breadth of the neutralizing response, we next determined the neutralizing activity against other variants: D614G, Alpha, Gamma, and Delta (Fig. 1F + G). Because plasma volumes were limited, neutralizing activity was only assessed when the mice were terminated (n = 10 two weeks after the second vaccination and n = 6 seven weeks after the second vaccination). Two weeks after the second vaccination, the neutralizing activity of Beta virosome-vaccinated mice was also significantly lower for D614G, Alpha and Delta (4.0-fold *p* = 0.0001, 3.6-fold *p* = 0.02 and 3.2-fold *p* = 0.007, respectively; Fig. 1F). Strikingly, Delta neutralization was undetectable in eight out of the ten Beta virosome-vaccinated mice and in three out of the ten Wuhan virosome-vaccinated mice. Gamma neutralization was not significantly different between the Wuhan and Beta vaccination groups (median ID_50_ 2139 and 1004, respectively, *p* = 0.25). Heterologous Beta neutralization in Wuhan virosome-vaccinated mice was also not statistically different from autologous neutralization in Beta virosome-vaccinated mice (median ID_50_ 648 and 729, *p* = 0.16). A similar trend was observed at seven weeks after the second vaccination, but because of the lower number of mice, no statistically significant differences were found (Fig. S1D). Of note, low level Delta neutralization (median ID_50_ between 119 and 464) was observed in four out of the six Beta virosome-vaccinated mice at seven weeks after the second vaccination, suggesting that Delta neutralization was improved in comparison to 5 weeks earlier. Altogether, we conclude that the neutralization breadth induced by Wuhan virosome vaccination is superior to that after Beta virosome vaccination.

To assess to which extent the neutralization response correlated with a general IgG response, we next measured IgG antibody responses using a custom Luminex assay^47^. IgG binding to both Wuhan and Beta spikes was determined in both vaccination groups at two weeks after the second vaccination (n = 16 per group). High IgG levels in both Wuhan virosome and Beta virosome-vaccinated mice were observed when autologous spike binding was assessed (median MFI 2363 and 2354, respectively; Fig. 1H + I). Similar to the neutralization results, IgG binding to Wuhan spikes was significantly reduced in Beta virosome-vaccinated mice (3.0-fold reduction, median MFI 2363 and 782, *p* = 0.0009), whereas median IgG binding to Beta spikes in Wuhan virosome-vaccinated mice was not significantly different from autologous binding to Beta spikes in Beta virosome-vaccinated mice (median MFI 1699 and 2354, *p* = 0.9). Median heterologous IgG binding to Alpha, Gamma and Delta spikes trended generally higher in Wuhan-vaccinated mice, but this was only significant for binding to the Alpha spike (median MFI 1743 and 868, *p* = 0.04). Of note, the reduction in Delta neutralization by Beta virosome-vaccinated mice seems more pronounced than the reduction in IgG binding to Delta spikes, suggesting that the induced antibodies against Delta are mainly non-neutralizing. Correlations between IgG and neutralizing antibody titers were generally weak and not statistically significant, with few exceptions such as for antibodies against the Wuhan variant (Fig. S1E).

To prevent infection, neutralizing antibodies need to be locally present at the site where the virus enters the human cells. To gain insight into the elicited immune response in the respiratory tract, we measured IgG2a antibody levels in broncheoalveolar lavage (BAL) at the time of sacrifice, i.e. two weeks or seven weeks after the second immunizations (Fig. S1F + G). Due to technical difficulties, we were unable to measure total IgG antibody levels. Generally, there were no differences between BAL IgG2a antibody levels. Only the binding to Delta spikes was significantly reduced by 2.1-fold in Beta-vaccinated mice in comparison to Wuhan-vaccinated mice (median MFI 390 and 185, *p* = 0.023) two weeks after the second vaccination, suggesting that Beta-vaccination also induces an inferior heterologous immune response in the respiratory tract.

The ongoing mass vaccination campaigns involving 6 billion vaccine administrations as of October 2021^48^, are cause for optimism that the SARS-CoV-2 pandemic will soon abate. However, the four original VOCs, Omicron, and yet to emerge SARS-CoV-2 strains pose a risk to the efficacy of current vaccines, as illustrated by the reduced performance of approved vaccines against Beta, Delta, and now Omicron^16–21, 49–54^. The emergence of neutralization resistant VOCs has therefore prompted the adaptation of vaccines to new variants and the assessment of mosaic vaccines that include both variant and Wuhan-based spike vaccines^25, 26, 55^. Generally, variant vaccines are expected to perform well in terms of autologous neutralization. However, neutralization of the Wuhan strain was reduced when a variant vaccine was used that contained the K417N, E484K, N501Y and D614G mutations^55^, which agrees well with our results. In fact, we show that our Beta virosome vaccine results in inferior neutralization breadth compared to the Wuhan virosome vaccine, indicating that in an unvaccinated population, a Beta-based vaccine is not a better choice in light of the currently circulating variants. Our study, which was performed and analyzed before the emergence of Omicron indicates that not all S proteins are equivalent when considering the induction of neutralization breadth. While the antigenically different Omicron likely warrants a vaccine update^56^, the induction of cross-neutralizing antibodies should be closely monitored.

Our study illustrates that the virosome platform should be further explored as a SARS-CoV-2 vaccination platform. While we assessed 3M-052 here as the built-in adjuvant, other adjuvants amendable for virosome incorporation such as the TLR2 agonist lipopeptide P3CK, the TLR4 agonist 6-acyl-PHAD, and the saponin QS21, could further tailor and enhance immune responses. Virosomes also form a promising platform for intranasal vaccinations^57^. Intranasal vaccine administration is non-invasive and may increase the willingness to be vaccinated. Especially in countries with a low COVID-19 vaccine acceptance, a non-invasive vaccine may be beneficial in reaching a sufficiently high vaccination rate.

In summary, we show that Beta spike virosomes induce potent neutralizing antibody responses against the Beta VOC, but that the neutralization breath of the response is inferior to that induced by Wuhan spike virosomes. These results are informative for guiding vaccine efforts to induce broadly neutralizing antibody responses against SARS-CoV-2.

## Materials and methods

### Protein design, expression and purification

Pre-fusion spike protein ectodomain DNA constructs were designed containing the following mutations compared to the Wuhan variant (Wuhan Hu-1; GenBank: MN908947.3): deletion of H69, V70 and Y144, N501Y, A570D, D614G, P681H, T716I, S982A and D1118H in Alpha; L18F, D80A, D215G, L242H, R246I, K417N, E484K, N501Y, D614G and A701V in Beta; L18F, T20N, P26S, D138Y, R190S, K417T, E484K, N501Y, D614G, H655Y and T1027I in Gamma. The constructs were ordered as gene fragments (Integrated DNA Technologies) and inserted into a pPPI4 expression vector containing a hexahistidine (his) tag using Gibson Assembly (ThermoFisher). All spike constructs were produced in HEK293F cells (ThermoFisher) and purified using NiNTA chromatography and size exclusion chromatography as previously described^58^. The Delta spike protein was provided by Dirk Eggink and Chantal Reusken (National Institute for Public Health and the Environment, the Netherlands).

### Virosomes

Virosomes were prepared as described earlier^44^. Briefly, inactivated influenza A/Brisbane/59/2007 (Seqirus, Australia) was solubilized with the detergent octaethyleneglycol-mono(n-dodecyl)ether (OEG) and the viral nucleocapsid was removed by centrifugation. To the supernatant, the lipids dioleoyl-phosphatidylcholine, cholesterol, and the click chemistry lipid dicyclobenzooctyl-phosphatidylethanolamine (DBCO-PE), dissolved in OEG, were added (all from Avanti Polar Lipids, USA). OEG was then removed by batch chromatography on BioBeads SM2 (BioRad, USA) as described^36^ and the virosomes were sterilized by filtration. 2-azidoethyl thiophosphodichlorate (ATPD) was synthesized and purified as described by Acme Bioscience (China)^59^. S protein was dialyzed against 50 mM HEPES pH 8.5 for 4 h and then mixed with ATPD at a 200:1 ratio of ATPD to protein for 1 h at RT. The product was dialyzed overnight against 2000 volumes of buffer (145 mM NaCl, 5 mM HEPES, 1 mM EDTA, pH 7.4). The resulting S-azide was filter-sterilized and incubated with virosomes for at least 24 h at 25 °C resulting in covalent coupling of S to virosomes through azide-DBCO-PE click chemistry. The concentration of S was estimated from SDS-PAGE gels. Adjuvants were inserted into the virosomal membrane by post-insertion. Briefly, 3M-052 (3M, USA) was dissolved in ethanol, a small quantity of this adjuvant was rapidly mixed with the virosomes and incubated for 30 min at RT. Coupling of virosomes to S was assessed by an ELISA in which antibody to the virosomal hemagglutinin was used to coat ELISA plates. After blocking, plates were incubated with virosomes and the presence of S in the virosomes was verified by using antibodies to S. During the ELISA, the virosomes remained intact.

### Mouse vaccinations

Female Balb/cAnNCrl mice received a subcutaneous prevaccination with inactivated influenza A/Brisbane/59/2007 virus into the neck skin-fold at week 0 to mimic an influenza-experienced immune system. At week 3 and 6, mice received a subcutaneous vaccination into the neck skin-fold with virosomes containing TLR7/8 agonist adjuvant 3M-052 and either Wuhan SARS-CoV-2 spike proteins or Beta spike proteins, n = 16 per group. Blood was collected at week 0, 6, 8 and 13. Ten mice per group were terminated at week 8, whereas the remaining six mice were terminated at week 13 to monitor the durability of the response. Mice were housed at the Animal Research Institute Amsterdam under BSL-2 conditions. All procedures were done in accordance with the Dutch Experiment on Animals Act and were approved by the Animal Ethics Committee of the Amsterdam UMC (Permit number 202011565) and in accordance with the ARRIVE guidelines.

### Luminex assays

A custom Luminex assay was used, as described previously^60^. In short, spike proteins were covalently coupled to Luminex Magplex beads with a two-step carbodiimide reaction at a ratio of 75 µg protein to 12.5 million beads. Following the outcome of optimization experiments, plasma samples were diluted 1:50,000 and BAL was diluted 1:10. Beads and diluted samples were incubated overnight, followed by detection with goat-anti-human IgG-PE (Southern Biotech). Read-out was performed on a Magpix (Luminex). Resulting mean fluorescence intensity (MFI) values are the median of approximately 50 beads per well and were corrected by subtraction of MFI values from buffer and beads only wells.

### SARS-CoV-2 pseudovirus neutralization assay

The pseudovirus neutralization assay was performed as described previously^45, 61^. Briefly, HEK293T cells expressing the SARS-CoV-2 receptor ACE2 were seeded in poly-L-lysine coated 96-wells plates and the next day triplicate serial dilutions of heat-inactivated serum samples were prepared, mixed 1:1 with SARS-CoV-2 pseudovirus, incubated for 1 h at 37 °C and then added in a 1:1 ratio to the cells. The pseudovirus titer of each variant was 1000 TCID50. After 48 h, the cells were lysed, transferred to half-area 96-wells white microplates (Greiner Bio-One) and Luciferase activity was measured using the Nano-Glo Luciferase Assay System (Promega) with a Glomax system (Turner Biosystems). Relative luminescence units were normalized to the units from cells infected with pseudovirus in absence of serum. Neutralization titers (ID_50_) were the serum dilution at which infectivity was inhibited 50%.

### Statistical analysis

*p* values below 0.05 were considered statistically significant. Mann–Whitney U-tests were used for unpaired comparisons, Wilcoxon matched-pairs signed rank tests were used for paired comparisons.

## Supplementary Information

Supplementary Information 1.

Supplementary Information 2.

## Data Availability

Data supporting the findings in this manuscript are available from the corresponding author upon request.
