# Supplementary figures and images for: A SARS-CoV-2 Wuhan spike virosome vaccine induces superior neutralization breadth compared to one using the Beta spike
Source: Sci Rep. 2022 Mar 10;12:3884. doi: 10.1038/s41598-022-07590-w (PMC8913678; doi:10.1038/s41598-022-07590-w)

A

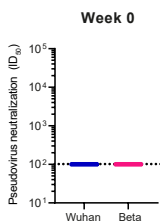

B

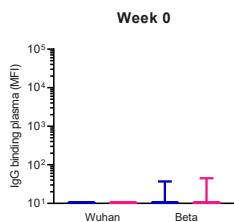

C

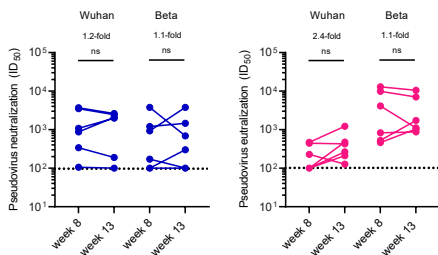

D

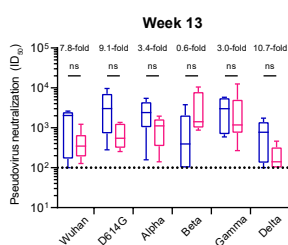

E

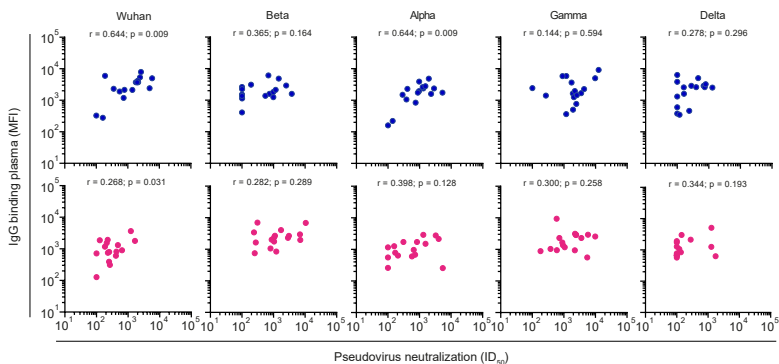

F

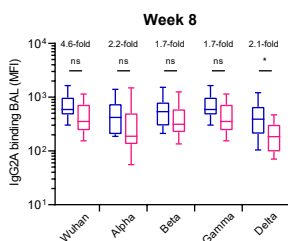

G

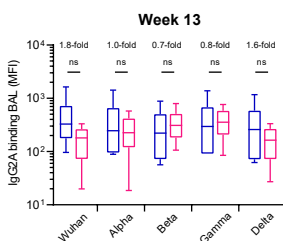

Supplement: Supplementary file 1 — Supplementary Information 1. [file 41598_2022_7590_MOESM1_ESM.pdf]
