# Supplementary material for: A SARS-CoV-2 Wuhan spike virosome vaccine induces superior neutralization breadth compared to one using the Beta spike
Source: Sci Rep. 2022 Mar 10;12:3884. doi: 10.1038/s41598-022-07590-w (PMC8913678; doi:10.1038/s41598-022-07590-w)
Supplement: Supplementary file 2 — Supplementary Information 2. [file 41598_2022_7590_MOESM2_ESM.docx]

**Supplementary Figure legend**

**Supplementary Figure 1. Binding and neutralizing antibody responses elicited by Wuhan and Beta spike virosome vaccines.**

(A) Autologous SARS-CoV-2 pseudovirus neutralization titers against Wuhan for Wuhan vaccinated mice and against Beta for Beta vaccinated mice at week 0 (before vaccination)(n = 16). (B) Anti-SARS-CoV-2 spike IgG levels against Wuhan and Beta spikes at week 0 (before vaccination) in both groups (n = 16 per group). (C) Paired comparison of autologous and heterologous SARS-CoV-2 pseudovirus neutralization titers at week 8 and week 13 (n=16 per group). (D) SARS-CoV-2 pseudovirus neutralization titers against Wuhan, D614G and all variants of concern at week 13 (n=6 per group). (E) Spearman correlations of IgG binding and neutralization in plasma against Wuhan and all variants of concern, week 8 and 13 combined (n = 16). (F) Anti-SARS-CoV-2 spike IgG2a levels in BAL measured by Luminex assay using beads coated with spike proteins of Wuhan and all variants of concern at week 8 (n=10 per group) and (G) at week 13 (n=6 per group). Wuhan-vaccinated animals are depicted in blue and Beta-vaccinated animals in magenta. Mann-Whitney U-tests were used for unpaired comparisons and Wilcoxon matched-pairs signed rank test for paired comparisons (∗p < 0.05, ns = not significant).
